# Indents

**Published:** 1902-04-15

**Authors:** 


### INDENTS.

☞ **Brief Articles of Technical or Scientific Value will receive notice and comment. Contributions invited.**

To Obviate Pain from the Hypodermic Puncture. —Make a preliminary puncture with a sterilized finely-pointed sewing needle.—*S. J. Spence.*

Removing the Gloss from Artificial Teeth.—Good effects may be had by etching the teeth for a few minutes in hydrochloric acid. They do not look well out of the mouth, but when wet with the saliva the appearance is very pleasing.—Dr. Werner, in *International.*

Orthoform for Exposed Pulps.—Introduce into the cavity a plug of cotton steeped in an alcoholic solution of orthoform. The pain will instantly cease and for a considerable time. This remedy is not poisonous and may be placed in the hands of patients.—*Med. Press.*

An antiseptic cavity varnish.—

℞ Select gum copal....℥ ss.
Ether...............℥ i.

dissolve and filter through paper and add

Hydronapthtol......gr. L.

and add enough ether to make the whole measure one ounce. Keep in well-stoppered bottles.—*Dental Era.*

To Anneal Steel.—Heat the steel slowly to the proper heat, a bright red, and quickly place it between two pieces of wood and screw them together in the jaws of a vise. The hot steel makes a charcoal bed for itself and is kept from the air until it becomes cold, when it will be found fairly soft and easy to work. Repeating the process will bring the most desirable qualities of softness.—*English Mechanic*.

A Varnish for Plaster Models which is superior to shellac or sandarac varnish is prepared as follows:

℞ Gum Sandarac........℥ ii.

Gum Mastix..........℥ i.

Venice Turpentine....℥ ss.

Alcohol..............℥ v.

Dissolve.

The varnish is colorless, elastic and leaves a fine glossy surface. Any alcohol-soluble anilin dye can be added to give the desired color.—*Dental Era*.

To Sterilize Dental Caries.—Clean thoroughly the cavity to be filled, washing the cavity several times with alcohol of 70, 90 and 100 degrees successively during a half a minute by means of a cotton pellet saturated with the alcohol. Then dry the cavity thoroughly with warm air and place in it a dressing of absolute alcohol, xylene, essence of geraneum and hydronaphtol, which should be left for twenty-four hours. After this treatment the permanent filling may be inserted.—J. Choquet, in *Cosmos*.

To Fill and Bake a Porcelain Inlay.—Hold the matrix firmly in pliers and drop a globule of mixed body into its center. By repeated rapping on the pliers settle the body solidly to place and get as dry as possible. The body can be so consolidated in this way as to prevent any considerable shrinkage when baked. Fill the matrix one-fourth full and bake nearly to a gloss; then add layer, after layer, baking each one until the proper contour is obtained. I use heavy platinum and do not invest for baking.—E. M. S. Fernandez, in *Review.*

Oxysulfate of Zinc Cement.—

| | | | |
|---|---|---|---|
| Powder: | ℞ | Calcined sulfate of zinc | ʒ. x. |
| | | Calcined oxid of zinc | ʒ. xv. |
| Liquid: | ℞ | Borax powder | gr. xv. |
| | | Gum arabic | ʒ. ii. |
| | | Water | ʒ. xxv. |

Filter.

This cement is a good substitute for Fletcher's artificial dentin and superior in many cases over temporary stopping, cotton-sandarac, etc. It is mixed in the same manner as oxyphosphate of zinc and becomes about as hard as well-set plaster. A filling of this cement will last in the mouth from two to three weeks.—*Dental Era.*

Chloroform for Children.—Mr. A. E. Francis read a report, published in the *Brit. Jour. Den. Sc.* of a case in which the administration of nitrous oxid to a girl ten years of age gave rise to "slow, shallow respiration, great lividity, and enormous venuous distention—the heart on auscultation and percussion being found to be distinctly distended and a systolic murmur being heard over the displaced apex. The recovery was good, and on a subsequent occasion when chloroform was used no trouble followed." This is another case in support of the opinion that chloroform is well tolerated by children.—*Digest.*

Gutta-Percha Separations.—A Chicago lady while visiting in Philadelphia called on a prominent dentist for service. Her teeth had a great many interproximal cavities, and the dentist used pink base-plate gutta-percha to secure the necessary separation. The lady was soon after taken sick and went to the seashore, returning direct to Chicago. By this time the separation between her teeth had become so great that her face was actually deformed by the protrusion of her front teeth. I removed the gutta-percha and filled the teeth, and they returned to their proper positions. I would warn you all not to leave gutta-percha between the teeth too long, thinking that it is not active.—Dr. Ames, in *Digest*.

GUM CHEWING; ITS EFFECT ON THE TEETH.—A. Lenhardtson, publishes in the *Med. Woche* a note upon the teeth of the inhabitants of the province of Dalecarlia in Sweden. He observed that 24.7 percent of the boys and 28 percent of the girls in the province of Goedermanland had caries of the permanent teeth, and that 38.5 percent of the boys and 34.2 percent of the girls had caries of the milk teeth; whereas in the province of Dalecarlia 15.6 percent of the boys and 16.2 percent of the girls had caries of the permanent teeth, and 34.4 percent of the boys and 42.1 percent of the girls had caries of the milk teeth. It will be noted that there was but little difference in the proportion of caries in the milk teeth of the children of both provinces. But in the permanent teeth the proportion of caries was very much lower in Dalecarlia than in Goedermanland. This comparative freedom from caries is attributed by the author to the practice of chewing burgundy pitch, which is universal in the province of Dalecarlia. The author furthermore attributes the beneficient action of the gum, not purely to its mechanical effect, but to the oils contained in it, which are of an antiseptic and bactericidal character.—*N. Y. Med. Jour.*

CROWNING A ROOT IN WHICH A POST HAS BEEN FRACTURED.—Choose a trephine into the hollow of which the fractured pin will fit without injury, and by means of the engine carry it as far up as is considered expedient. Around the end of the fractured pin and into the groove made by the trephine a tube of English dental alloy is fitted, and soldered to a cap and band, as in an ordinary Richmond crown. This device I have found to give the most secure grip with a minimum sacrifice of tissue. It It will be noted that such a tube, band and cap, afford really a triple grip. First, the tube grasps the broken pin ; second, the tube is held externally by the surrounding dentin ; and, third, the circumferential band secures both. It will be obvious that such a tube is immeasurably better than a solid post of equal length, the latter being held only externally. The tube, further ; need not to be inserted over the fractured pin to any great depth, as would be neccessary where a solid pin is used. Its tight encircling of its broken predecessor makes it almost continuous with it and enables it to utilize the anchorage of the whole length of the original post. The stability to be obtained from a tube only one-eight of an inch long will be found, to those who have not tried it, to be perfectly surprising.—J. Girwood, in *Cosmos*.

A RAPID METHOD OF PRODUCING REGULATING PLATES

or Partial Dentures in Hard Rubber.—Mr. Geo. Brunton (*Journal of the British Dental Association*) is the author of the following description of a rapid method of making rubber plates. The plaster model must be dry. The surface to be covered by the plate is first painted with a solution of rubber in chloroform (a good rubber for this purpose is the quick vulcanizing rubber). The chloroform is then allowed to evaporate, which will take place in a few minutes. A piece of quick vulcanizing base-plate rubber cut somewhat near the size required is warmed and pressed down on the painted surface of the model, taking care that it comes in close contact with every part. The superfluous edges are then trimmed off and the regulating appliances put in position, or the teeth are mounted by warming them and pressing them into their places. At this stage of the operation the rubber is covered with tin foil and placed in a metal box of any kind large enough to hold the model, which is then surrounded with French chalk (steatite) which has to be packed lightly so as not to disturb the teeth; the lid is fastened down with wire or cord and the piece is then vulcanized in the ordinary way. The advantages of this method are the saving of time, no investment in plaster of Paris or a flask being necessary, and the production of a superior quality of rubber.

